# SMG-BERT: integrating stereoscopic information and chemical representation for molecular property prediction

**DOI:** 10.3389/fmolb.2023.1216765

**Published:** 2023-06-30

**Authors:** Jiahui Zhang, Wenjie Du, Xiaoting Yang, Di Wu, Jiahe Li, Kun Wang, Yang Wang

**Affiliations:** ^1^ School of Software Engineering, University of Science and Technology of China, Hefei, China; ^2^ Suzhou Institute for Advanced Research, University of Science and Technology of China, Suzhou, Jiangsu, China; ^3^ School of Computer Science and Technology, University of Science and Technology of China, Hefei, China

**Keywords:** molecular property prediction, chemical feature fusion, unambiguous molecular descriptor, molecular representation learning, molecular stereoscopic information

## Abstract

Molecular property prediction is a crucial task in various fields and has recently garnered significant attention. To achieve accurate and fast prediction of molecular properties, machine learning (ML) models have been widely employed due to their superior performance compared to traditional methods by trial and error. However, most of the existing ML models that do not incorporate 3D molecular information are still in need of improvement, as they are mostly poor at differentiating stereoisomers of certain types, particularly chiral ones. Also,routine featurization methods using only incomplete features are hard to obtain explicable molecular representations. In this paper, we propose the Stereo Molecular Graph BERT (SMG-BERT) by integrating the 3D space geometric parameters, 2D topological information, and 1D SMILES string into the self-attention-based BERT model. In addition, nuclear magnetic resonance (NMR) spectroscopy results and bond dissociation energy (BDE) are integrated as extra atomic and bond features to improve the model’s performance and interpretability analysis. The comprehensive integration of 1D, 2D, and 3D information could establish a unified and unambiguous molecular characterization system to distinguish conformations, such as chiral molecules. Intuitively integrated chemical information enables the model to possess interpretability that is consistent with chemical logic. Experimental results on 12 benchmark molecular datasets show that SMG-BERT consistently outperforms existing methods. At the same time, the experimental results demonstrate that SMG-BERT is generalizable and reliable.

## 1 Introduction

The prediction of molecular properties is one of the fundamental tasks in chemistry (Wieder et al., 2020) and deserves special attention. Traditional computational methods, such as density functional theory (DFT) or field experiments, are time-consuming and poorly scalable with size (Chen et al., 2021). This could cause inevitable and serious moral and ethical issues with experimental testing involving animals or humans *in vivo*. Recently, Machine Learning (ML), including Deep Learning (DL), has emerged as a powerful data-driven approach for establishing a connection between molecular structure and properties (Chen et al., 2021). ML methods can often deliver results that are comparable to DFT in terms of accuracy while being significantly faster by approximately 3-5 orders of magnitude (Hohenberg and Kohn, 1964; Kohn and Sham, 1965).

A key component/challenge in applying ML to molecular science is molecular featurization. This transforms molecular structures into machine-readable formats (Wu et al., 2018) and therefore dictates the embedded chemical information into final representations (Raghunathan and Priyakumar, 2021). Effective molecular representations are essential for a variety of molecular prediction tasks, such as property prediction (Du et al., 2023a), retrosynthesis (Segler et al., 2018; Zhang et al., 2022), generative molecular design (Moret et al., 2020), and so on (Dral and Barbatti, 2021). Current molecular representations can be categorized into three different classes: molecular fingerprints based on molecular topological substructures encoded as a sequence of bits, sequence-based representations by SMILES, and graph-based representations (Fang et al., 2022). However, current featurization methods still have certain shortcomings, as they only focus on extracting various hierarchical molecular information, which makes it challenging to thoroughly integrate the molecular information and achieve effective generalization among potential chemical compounds. In this study, one-dimensional (1D) SMILES strings, two-dimensional (2D) topological structures, and three-dimensional (3D) geometric structures are the intuitive expressions of molecular information at different levels. SMILES strings could naturally be used as input to some NLP models such as Transformer (Tetko et al., 2020; Schwaller et al., 2021) and BERT (Wang et al., 2019; Zhang et al., 2021) to reach high performance, no matter if for a molecular generation (Moret et al., 2020) or property prediction (Chen et al., 2021; Du et al., 2023a), However, these methods tend to lose the chemical context during preprocessing, as they often remove essential chemical symbols such as “#” and “( )”, from the SMILES string. Moreover, only 1D information would inevitably lose adjacency information (Du et al., 2023b). The 2D topological structure is one of the most important chemical representations, which was expertly developed and has been used for centuries as a crucial carrier for the exchange, dissemination, and transmission of chemical knowledge. However, it is difficult to distinguish stereochemistry molecular features such as cis-trans isomerism, chirality, and other enantiomers only based on adjacency matrices (Stärk et al., 2021; Fang et al., 2022). Therefore, 3D information is an important and non-negligible piece of knowledge that the model needs to master to solve stereochemical problems (Chen et al., 2021; Du et al., 2023b). Each of these three modalities focuses on different aspects, and all are fundamental to molecular featurization.

On the other hand, interpretability is also an obstacle to the widespread application of deep learning models. Current ML models mainly focus on the prediction task of compound properties, but only a few ML methods are interpretable (Wang et al., 2021). Therefore, there is often a trade-off between predictive performance and the ability to interpret ML models (Rodriguez-Perez and Bajorath, 2021). Although causal analysis theories such as contrastive explanations or counterfactuals, feature perturbation (sensitivity analysis), and gradient-based methods could obtain feature importance analysis to a certain extent, interpretable results still need to be improved to match the actual chemical logic for individual explanations (Prosperi et al., 2020; Wang et al., 2021). Attention mechanisms have been widely adopted for visualizing molecular prediction results, as they allow for intuitive visualization and human-friendly explanations (Ross et al., 2022). However, to the best of our knowledge, current attention mechanisms rarely embed basic chemical intuitions or expert prior knowledge to enhance interpretability. Chemical properties are ultimately determined by intrinsic properties (Zhang et al., 2022), and most of these are determined by the electron density and electronegativity of neighboring atoms, which could be represented by NMR chemical shifts and bond dissociation energy (BDE). Thus, we could consider them perfect candidates for ML descriptors to improve model interpretability.

In this paper, we propose a stereo molecular graph BERT (SMG-BERT) by integrating the 3D space geometric parameters, 2D adjacency information, and 1D SMILES representation into a self-attention-based BERT model. SMG-BERT could generate accurate chemical representations for various molecules, including chiral molecules, which provides assurance for precise property prediction results and expands the application scope. Meanwhile, SMG-BERT incorporates the NMR chemical shifts and bond dissociation energies (BDEs) as chemical descriptors using a transformer encoder to improve interpretability. This results in visualizations that conform to chemical logic and are more convincing. A series of experimental results show that SMG-BERT can consistently outperform previous state-of-the-art molecular property prediction models on 12 benchmark molecular datasets.

## 2 Methods

In this section, we describe in detail the data preprocessing process, model structure, and loss function in three parts. In the data preprocessing process, the model could obtain an input representation that consists of three components: a molecular representation z is generated solely from the atomic and NMR sequence by the embedding layer, which lacks topological information and thus can be regarded as 1D information. The bond dissociation energy matrix B , which not only provides topological information but also includes vital chemical knowledge about bond energies. Finally, the distance fraction matrix D , based on the distance matrix $D^{raw}$ , could be regarded as 3D information. We present the implementation details of our model architecture, which is based on the transformer-encoder architecture and introduces multiple modal information of the molecules. Meanwhile, various learning tasks are presented in the pre-training phase to enhance the representation capabilities of the model.

### 2.1 Data preprocessing

In the pre-training process, the dataset was collected from PubChem (Kim et al., 2023). Although increasing the amount of pre-training data could potentially further improve the performance of the model, the improvement in model performance became less significant after a 480 k training size (Supplementary Figure S1). Considering the balance between training time and effect, we randomly selected 480 k molecules (SMILES). Three preprocessing tasks were performed, including generating: (1) input representation *z* of the molecules (2) the bond dissociation energy matrix *B*,(3) and the distance fraction matrix *D*.

**The input representation** z **of the molecules:** we used RDKIT to transform each SMILES into an atomic sequence $S_{A}=\left[A_{1},A_{2},\cdots ,A_{n}\right]$ of length n and generate the corresponding NMR sequence $S_{N}=\left[N_{1},N_{2},\cdots ,N_{n}\right]$ for the atomic sequence $S_{A}$ by a DL model with six message-passing neural networks (MPNN) layers and two fully connected network layers as in our previous work (Zhang et al., 2022) (continuous NMR was transformed into discrete). Then, 80% of Atom/NMR in the two sequences were randomly selected and replaced by <M> (which stands for MASKL); 10% were replaced by another Atom/NMR one, and the rest were left unchanged. In addition, we added a global node <G> at the beginning of the sequence, which represents the global representation of the whole molecule. Finally, two independent embedding layers were used to map the two new input sequences $S_{A}^{\prime }$ and $S_{N}^{\prime }$ to a continuous input representation $z=\left[z_{1},z_{2},\cdots ,z_{n}\right]$ :

$$z=E_{A}\;\left(S_{A}^{\prime }\right)∥E_{N}\;\left(S_{N}^{\prime }\right)$$

where $E_{A}$ is the embedding layer of the atomic sequence, $E_{N}$ is the embedding layer of the NMR sequence, and ∥ denotes the concatenation operation.

**The bond energy matrix:** we generated the bond energy matrix B by an additional DL model with four MPNN layers and two fully connected network layers according to the method in our previous work (Zhang et al., 2022), and normalized it:

$$B^{norm}=Norm\left(B\right)=\frac{B-B_{\min}}{B_{\max}-B_{\min}}$$

where $B_{\max}$ is the maximum value of matrix B and $B_{\min}$ is the minimum value of matrix B .

**Distance fraction matrix** $D^{\prime }$ **:** the ground state 3D structure of the molecule can be obtained by the RDIKT package. Based on this, we were able to obtain the atomic distance information and generate the original distance matrix $D^{raw}.$ Then, the distance matrix $D^{raw}$ was transformed by a transformer encoder layer into the distance fraction matrix D .

$$D=Trans\left(D^{raw}\right)$$

where $D^{raw}$ represents the 1,2,⋯,n− th column vector of $D^{raw}$ , and Trans is a transformer encoder module.

### 2.2 Modified attention mechanism

Our model is based on the self-attention mechanism. For our task, the input representation z was first mapped onto the query matrix Q , the key matrix K , and the value matrix V using the projection matrices $W_{q},W_{k},W_{v}$ , respectively:

$$Q=W_{q}z$$

$$K=W_{k}z$$

$$V=W_{v}z$$

The attention score matrix A could then be calculated from the Q,K matrix. Specifically, we computed the dot products of the query with all keys, divided each by $d_{k}$ , and applied a softmax function to obtain the weights on the values.

$$A=softmax\left(\frac{QK^{T}}{\sqrt{d_{k}}}\right)$$

where $d_{k}$ is the dimension of the key.

However, the global attention score matrix, A , is difficult to optimize because it requires considering the relationships among all the atoms, resulting in a high degree of freedom. To address this problem, we introduced an adjacency matrix to constrain the global attention score matrix:

$$M=Binary\left(B\right)$$

$$A_{2d}=A⊙M+\lambda Norm\left(B^{norm}\right)$$

where *"* Binary” is a binarization operation that transforms the bond-energy matrix into an adjacency matrix M , ⊙ denotes an element-wise product, and λ is a balancing hyperparameter between the mask attention score matrix and the bond-energy matrix. Here, λ is set to 0.2. The hyperparameters are provided in Supplementary Tables S1, S2.

Furthermore, to incorporate 3D information, we brought the distance matrix *D* into the attention score matrix to reflect the interaction strength of atoms:

$$A_{3d}=A_{2d}+D$$

Once the final correlation matrix $A_{3d}$ is obtained, we multiplied it with the value matrix V to obtain the output sequence z :

$$z=A_{3d}V$$

In addition to the attention sub-layers, the transformer encoder layer also contains a position-wise feed-forward network:

$$r_{i}=FFN\left(z_{i}\right)$$

where $r_{i}$ denotes the final output representation of the i− th atom. We wrote the representation of the whole sequence of atoms as $r=\left[r_{1},r_{2},\cdots ,r_{n}\right]$ .

### 2.3 Loss function

During the pre-training stage, we aimed to increase the richness of information contained in the atomic representation sequence r . To achieve this, we propose three self-supervised learning (SSL) tasks: atomic and NMR reconstruction, bond energy prediction, and 3D information reconstruction.

**Atomic and NMR reconstruction**: During data preprocessing, some atoms in the atomic sequence are randomly replaced by the special token "<M>". The task of atomic reconstruction involves predicting the correct class of these masked atoms. Specifically, given the representation $r_{i}$ of the masked atom, the model outputs the predicted class probability $p_{i}$ after passing through the MLP and SoftMax layers.

$$p_{i}=softmax\left(MLP\left(r_{i}\right)\right)$$

The cross-entropy loss is used as the loss function, which computes the difference between the predicted probability $p_{i}$ and the ground truth label $y_{i}$ of the masked atom:

$$L_{A}=-\frac{1}{m}\sum_{i=1}^{m}y_{i}\log p_{i}$$

where m is the total number of masked atoms.

Similarly, the NMR reconstruction task is consistent with the atomic reconstruction principle, which we denoted as $L_{N}$ .

**Bond energy prediction:** The bond representation can be determined by the nodes connected at both ends. The predicted bond energy $q_{ij}$ between the atomic representation $r_{i}$ and $r_{j}$ can then be obtained by running the bond representation through the MLP.

$$q_{ij}=MLP\left(r_{i}∥r_{j}\right)$$

where ∥ denotes the concatenation operation. Mean Squared Error (MSE) is the loss function and $y_{ij}$ is the ground truth:

$$L_{B}=\sum_{i=1}^{n}\sum_{j=1}^{n}{\left(y_{ij}-q_{ij}\right)}^{2}$$

**3D information reconstruction**: To avoid the complexity of modeling direct prediction of atomic coordinates, which requires translation-rotation invariance and order invariance, we use intermediate quantities that reflect 3D information, such as interatomic distances, bond angles, and torsion angles, to predict atomic coordinates. Specifically, the atomic representation r is mapped to a new representation $r^{\prime }$ using the projection matrix $W_{r}$ , with a vector length of 3 to represent the coordinates in 3D space.

$$r^{\prime }=W_{r}r$$

The interatomic distances $\hat{d}$ , bond angles $\hat{\theta }$ , and torsion angle $\hat{\varphi }$ predicted by the model can be calculated directly:

$$\hat{d}={\left\|r_{i}^{\prime }-r_{j}^{\prime }\right\|}_{2}$$

$$\hat{\theta }=\cot^{-1}\left(\frac{r_{i}^{\prime }\cdot r_{j}^{\prime }}{<r_{i}^{\prime },r_{j}^{\prime }>}\right)$$

$$\hat{\varphi }=\cos^{-1}\left(\frac{n_{\alpha }\cdot n_{\beta }}{\left|n_{\alpha }\right|\cdot n_{\beta }}\right)$$

where i and j refer to two different atoms, $r_{i}^{\prime }$ and $r_{j}^{\prime }$ indicate the coordinate vectors of atoms i and j , $n_{\alpha }$ and $n_{\beta }$ correspond to the normal vector of the α and β planes.

Finally, we used the mean squared error (MSE) as the loss function to compute the difference between the predicted values and the corresponding ground truth values for atomic distances d , bond angles θ , and torsion angles φ .

$$L_{3D}={\left(d-\hat{d}\right)}^{2}+{\left(\theta -\hat{\theta }\right)}^{2}+{\left(\varphi -\hat{\varphi }\right)}^{2}$$

**Loss functions**: To balance the different objective functions represented by $L_{A},L_{N},L_{B}$ , and $L_{3D}$ , it is necessary to consider their relative importance. The σ1, σ2, σ3, and σ4 are the learnable parameters as the proportion of $L_{A},L_{N},L_{B}$ , and $L_{3D}$ in the total loss (Kendall et al., 2017), and are optimized through backpropagation to appropriate values. This enables the model to effectively learn from all four SSL tasks while ensuring that the different losses are appropriately weighted.

$$L=\frac{1}{\sigma_{1}^{2}}L_{A}+\frac{1}{\sigma_{2}^{2}}L_{N}+\frac{1}{\sigma_{3}^{2}}L_{B}+\frac{1}{\sigma_{4}^{2}}L_{3D}+\log\sigma_{1}+\log\sigma_{2}+\log\sigma_{3}+\log\sigma_{4}$$

### 2.4 Baseline model and test data sets

Several advanced models in recent years were selected for comparison as the baseline, namely, GAT (Veličković et al., 2017), GIN (Xu et al., 2018), D-MPNN (Yang et al., 2019), GROVER (Rong et al., 2020), GraphMVP (Liu et al., 2021a), and AttentiveFP (Xiong et al., 2019). Among them, GIN, GAT, D-MPNN, and AttentiveFP are all non-pre-training methods based on GNN. GAT introduced the attention mechanism into GNN and adaptively learned the weight of nodes. GIN was derived from the Weisfeiler-Lehman graph isomorphism test degree and exhibited almost the same representation ability as the WL test D-MPNN utilizes messages that are associated with directed edges (bonds) rather than atom nodes. AttentiveFP presents a novel graph neural network architecture that incorporates an attention mechanism to extract nonlocal effects at the intramolecular level for molecular representation. GROVER and GraphMVP employ a pre-training process. GROVER can effectively learn rich structural and semantic information about molecules from a large volume of unlabeled molecular data by performing SSL tasks at the node, edge, and graph levels. Meanwhile, GraphMVP uses an SSL approach to achieve correspondence and consistency between 2D topological structures and 3D geometric views.

A total of 12 datasets (seven for regression and five for classification) were selected from MoleculeNet (Wu et al., 2018) and ADMETlab (Dong et al., 2018) to conduct downstream experiments. According to this benchmark (Rong et al., 2020; Liu et al., 2021b), we split these datasets with scaffolds according to the molecular substructure, as this splitting method is more challenging and better evaluates the generalization ability in out-of-distribution data. In the testing process, we randomly selected 80% of the samples as the training set, 10% as the validation set, and the remaining 10% as the test set. Five independent runs were executed for each method, and the mean and standard deviation of the metrics were reported. ROC-AUC, RMSE, and *R* ^2^ are used as evaluation indicators for classification and regression tasks, respectively.

## 3 Results and discussion

### 3.1.1 Model architecture of SMG-BERT

**FIGURE 1:**
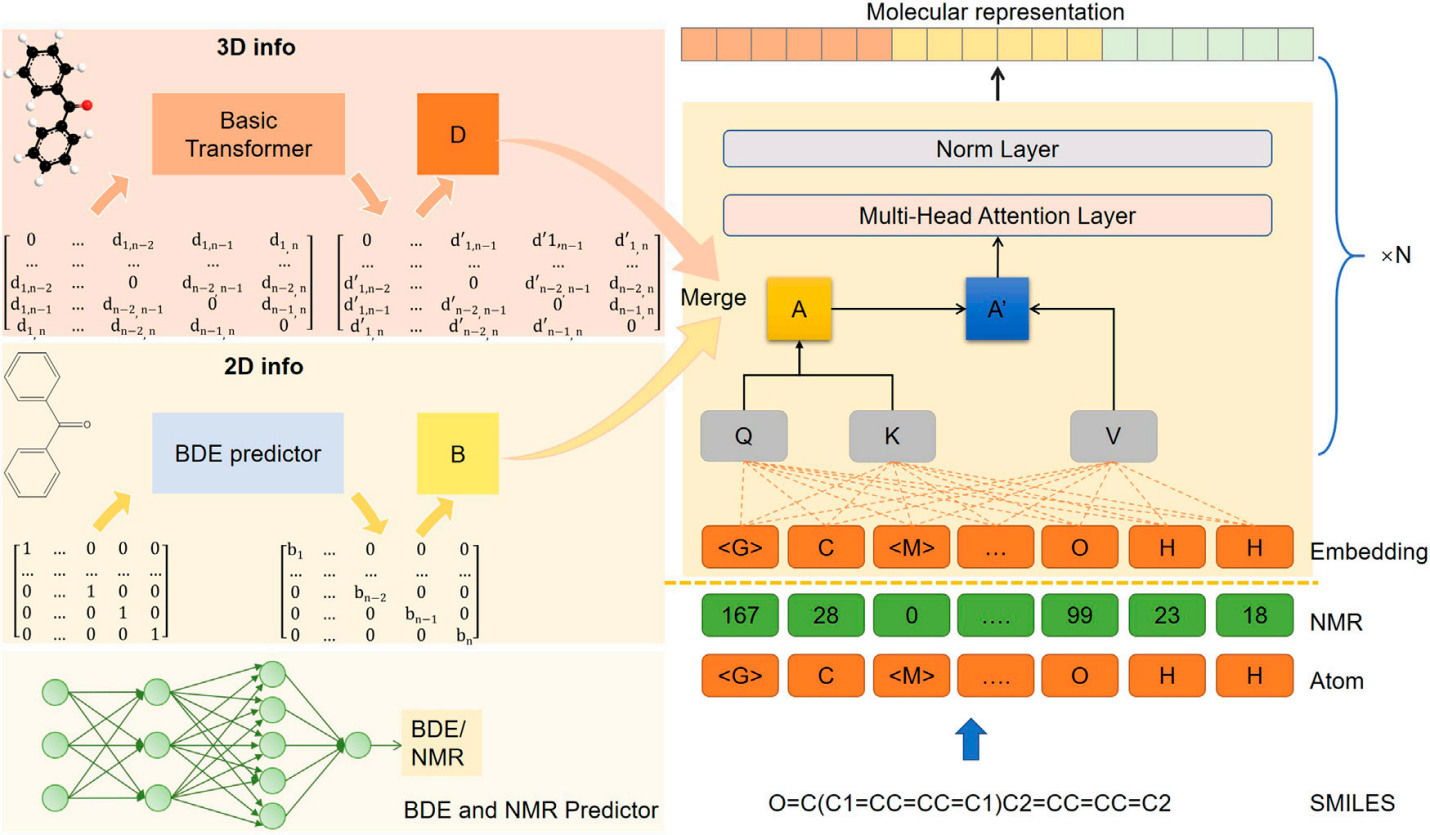
The model architecture of SMG-BERT. 2D topology graphs and 3D ground state conformations are generated by SMILES. A is an attention score matrix and B is a binding energy matrix. D is an adjusted distance matrix between atomic pairs by a basic transformation model.

The architecture of our model is shown Figure 1, consisting of one embedding layer, six transformer encoder layers, and one output layer. The model processes 1D, 2D, and 3D information separately. The 1D information includes both the atomic sequence obtained from the SMILES string using the RDKIT package (Landrum, 2019) and the NMR sequence generated (Zhang et al., 2022) (the predicted NMR values are discretized). Each sequence is independently masked by about 20% (as a hyperparameter) and then embedded in a high-dimensional vector space through two separate embedding layers. For the 2D information, we introduced the bond energy result (B matrix in Figure 1) to provide differentiation information about the bond connection. The B matrix is fused into the global attention score matrix (A matrix in Figure 1) at the transformer encoder layer. As for the 3D geometric information, we calculated the interatomic distances, bond angles, and torsion angles in the ground state conformations using the RDKIT package (Faber et al., 2017; Lubbers et al., 2018). The distance matrix was then processed by an additional transformer encoder module to obtain the distance fraction matrix (D matrix in Figure 1) as the final 3D information, where the farther distance could have a smaller value. These three modal inputs, along with multiple self-supervised learning tasks, which include masked atom inference and 3D geometric feature reconstruction, allow for a multimodal representation of model learning.

The resulting molecular representation would be used for downstream tasks and would adopt the fine-tuning method. Specifically, after pre-training, the atom representation of the global super-node “<G>” is the final molecular representation, with a 512-dimensional vector. This would be fed into a two-layer, fully connected network with random initialization, which yields the final prediction results. The network uses ReLU as the activation function and sets the dropout ratio to 0.1. Considering that catastrophic forgetting issues could occur as the model targets specific downstream tasks that are completely different from the pre-training process (Kirkpatrick et al., 2017), we would retain the pre-training loss as a regular term, which would maintain the chemical information and spatial characteristics learned in the pre-training process In addition, our model is a flexible, comprehensive feature fusion framework that supports multi-dimensional information removal and fusion. For specific downstream tasks, 3D or chemical information could be considered a super parameter, and we could dynamically adjust or increase the available input features according to the target.

### 3.2 Model validation results on common datasets

Table 1 shows that compared to no pre-training, the RMSE index decreased by 12.71%, while the ROC-AUC improved by 20.7% on the classification task. And *R* ^2^ increased by 5.07% in Supplementary Table S3. These results demonstrate the importance and necessity of pre-training in our strategy. Moreover, a noteworthy trend is that the smaller the dataset, such as FreeSolv and ESOL, the higher the improvement effect to some extent, which demonstrates the excellent generalization ability of the pre-trained model. Besides, Table 1 also records the prediction results and the performance of our model with several advanced models. SMG-BERT outperforms six out of eight baselines and achieves a close second in the other two (Tox21 and HIV). Specifically, in all four regression datasets, SMG-BERT achieves the SOTA results and has an overall relative improvement of 15.3% on average compared to previous SOTA results. Relatively, only 5.81% is achieved on average for the AUC-ROC score in classification tasks, which could be due to the regression tasks being more relevant to the 3D geometric information of molecules (Fang et al., 2022), such as the label of water-soluble or hydration-free energies in ESOL and FreeSolv dataset, which is closely related to the molecular polarity, which is in turn the geometric symmetry concept of the 3D conformation of a molecule. Especially on the QM7, QM8, and QM9 datasets, the improvement results are more significant, reaching an average of 20.7%. The properties in these datasets are directly related to the 3D geometric information.

**TABLE 1:** Overall performance for regular regression and classification tasks.

Regression								Classification				
Dataset	ESOL	FreeSolv	Lipo	LogS	QM7	QM8	QM9	BACE	Tox21	HIV	BBBP	BBBP
NO. molecules	1,128	642	4,200	5,045	6,830	21,786	133,885	1,513	7,831	41,127	2039	2039
GIN	0.982_(0.049)_	2.023_(0.036)_	0.723_(0.038)_	1.739_(0.123)_	94.7_(4.32)_	0.0193_(0.0011)_	0.00923_(0.00007)_	0.752_(0.027)_	0.768_(0.008)_	0.727_(0.013)_	0.663_(0.021)_	0.663_(0.021)_
GAT	1.433_(0.078)_	2.317_(0.077)_	1.054_(0.056)_	1.675_(0.166)_	84.6_(3.98)_	0.0182_(0.0009)_	0.00868_(0.00012)_	0.771_(0.015)_	0.755_(0.006)_	0.746_(0.007)_	0.641_(0.032)_	0.641_(0.032)_
D-MPNN	0.988_(0.010)_	1.889_(0.042)_	0.732_(0.053)_	1.302_(0.084)_	101.6_(4.32)_	0.0201_(0.0007)_	0.01023_(0.00004)_	0.799_(0.025)_	0.750_(0.060)_	**0.769**_(0.009)_	0.712_(0.024)_	0.712_(0.024)_
AttentiveFP	0.865_(0.066)_	1.891_(0.063)_	0.710_(0.012)_	1.226_(0.066)_	69.3_(3.78)_	0.0204_(0.0008)_	0.00873_(0.00004)_	0.792_(0.024)_	0.765_(0.007)_	0.760_(0.006)_	0.721_(0.017)_	0.721_(0.017)_
GROVER	0.973_(0.042)_	1.826_(0.101)_	0.766_(0.033)_	1.214_(0.032)_	91.3_(3.29)_	0.0211_(0.0014)_	0.00802_(0.00005)_	0.812_(0.016)_	0.749_(0.004)_	0.701_(0.011)_	0.701_(0.013)_	0.701_(0.013)_
GraphMVP	0.947_(0.020)_	1.841_(0.054)_	0.718_(0.033)_	1.163_(0.073)_	98.4_(4.20)_	0.0208_(0.0018)_	0.00899_(0.00007)_	0.819_(0.017)_	**0.772**_(0.003)_	0.743_(0.007)_	0.722_(0.016)_	0.722_(0.016)_
Our method (no PT)	0.974_(0.033)_	1.893_(0.063)_	0.756_(0.033)_	1.295_(0.033)_	86.3_(3.78)_	0.0193_(0.0012)_	0.00942_(0.00006)_	0.660_(0.039)_	0.764_(0.008)_	0.710_(0.016)_	0.649_(0.022)_	0.649_(0.022)_
Our method (PT)	**0.859**_(0.029)_	**1.616**_(0.047)_	**0.694**_(0.033)_	**1.120**_(0.052)_	**57.4**_**(3.01)**_	**0.0172**_**(0.0008)**_	**0.00792**_**(0.00004)**_	**0.823**_(0.012)_	0.766_(0.008)_	0.758_(0.007)_	**0.736**_(0.014)_	**0.736**_(0.014)_

ROC-AUC was used for classification tasks, and RMSE was used for regression tasks, with standard deviations in brackets; PT, pre-training. Bold numbers indicate the best result. Standard deviations are in brackets.

Bold numbers indicate the best result.

**FIGURE 2:**
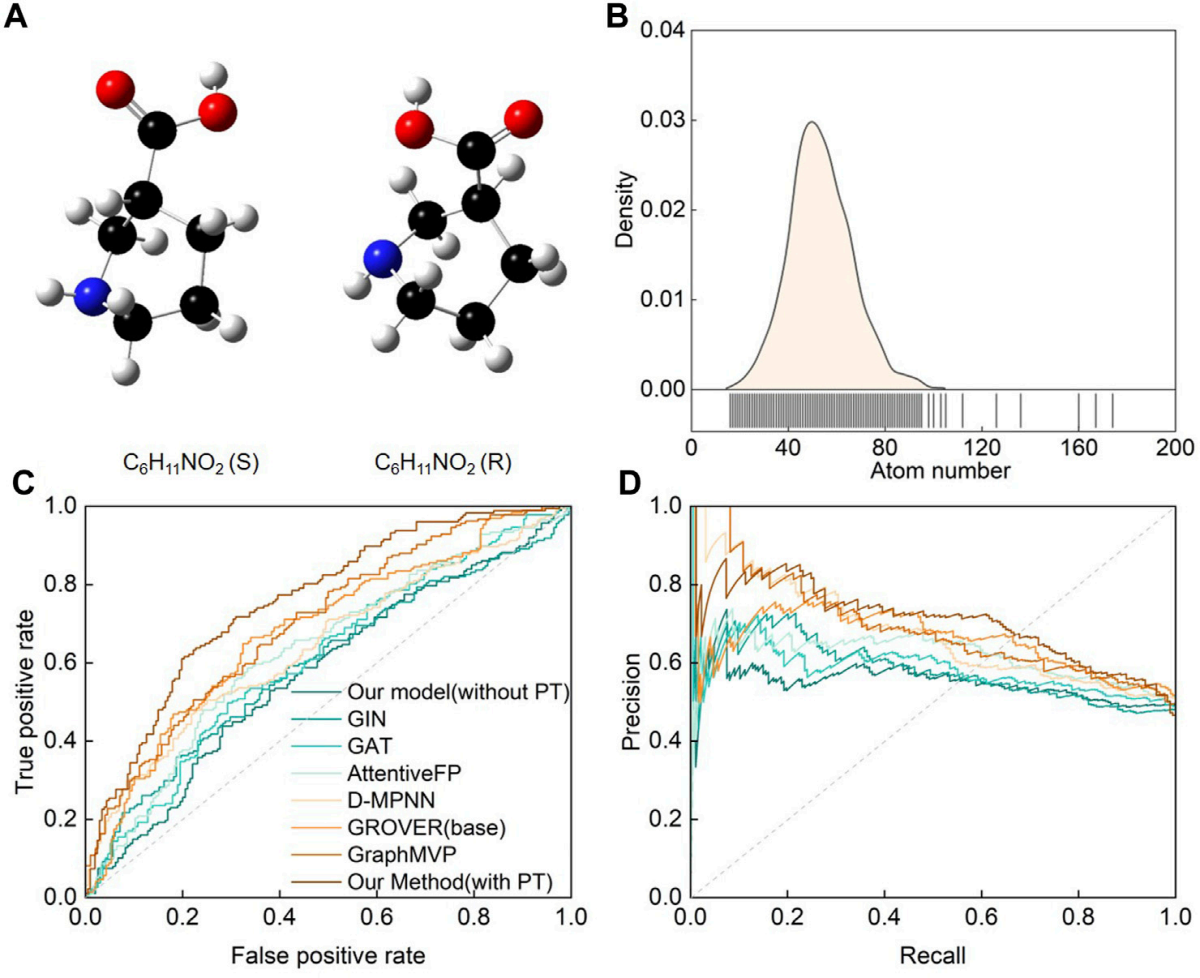
Performance of the SMG-BERT model in discriminating chiral molecules. **(A)** A pair of chiral molecules of L-proline and R-proline as an example. **(B)** Atom count distribution of the chiral molecule dataset. **(C)** ROC and **(D)** PRC curves of CFFN compared with other random classification models in discriminating enantiomeric pairs. PT: Pre-training.

On the other hand, stereochemical molecules deserve our special attention because they are a rarely studied class of molecules in nature. Current DL models often overlook chiral pair discrimination, leading to inaccurate predictions (MacKenzie and Stachelek, 2021; Cho et al., 2023). Although chiral analysis is fundamental to many fields, limited datasets restrict our ability to study it. Nonetheless, we conducted a macromolecule chiral classification task to evaluate SMG-BERT’s prediction and generalization capabilities. A protein-chiral ligand binding dataset was used in this case, where each enantiomer of the ligand could demonstrate significantly different binding affinities. In this dataset, a chiral pair was defined as two enantiomers measured in the same biochemical binding assay, which is a common occurrence in biochemistry referred to as a “chiral cliff” (Schneider et al., 2018) (Figure 2A). The dataset contained approximately 3,800 chiral pairs with a more complex structure that included a diverse range of atoms and elements, such as C, H, O, N, B, Br, Cl, and so on (Figure 2B).

This dataset was divided into training, validation, and test sets in a ratio of 8:1:1. As shown in Figure 2C, SMG-BERT could effectively discriminate between chiral molecules, achieving an AUC score of 0.75, which is about 12.81% higher than the other models on average. The PRC curve also shows that our model outperformed the other models (Figure 2D). Obviously, including 3D geometric information models such as GraphMVP or GROVER is better than using models based on 2D molecular graphs since the left- and right-handed versions of enantiomers have identical connectivity (Du et al., 2023b). Additionally, as we can see, without the pre-training process, the classification accuracy of the model would drop significantly, approaching 50%. This level of accuracy is virtually meaningless, given that the problem is a binary classification task. 3D information is relatively difficult to capture and is especially important in 3D-related downstream tasks. During pre-training, our model focuses on learning the complete 3D stereo geometric information of the molecules by incorporating interatomic distance, angle, and dihedral angle, which is a critical factor contributing to the superiority of our model over other models. In addition, the explicitly introduced distance information is also more conducive to the interpretability of the model and better reflects the correlation between the atoms.

### 3.3 Interpretability analysis

In the final phase of our study, we examined the attention matrix generated by SMG-BERT to reveal the chemical insight acquired during pre-training. We calculated the similarity between attentional scores for atoms at different levels of information integration, using the benzophenone molecule (C_15_H_12_O) as a case study. We also presented visualization results for several molecules.

**FIGURE 3:**
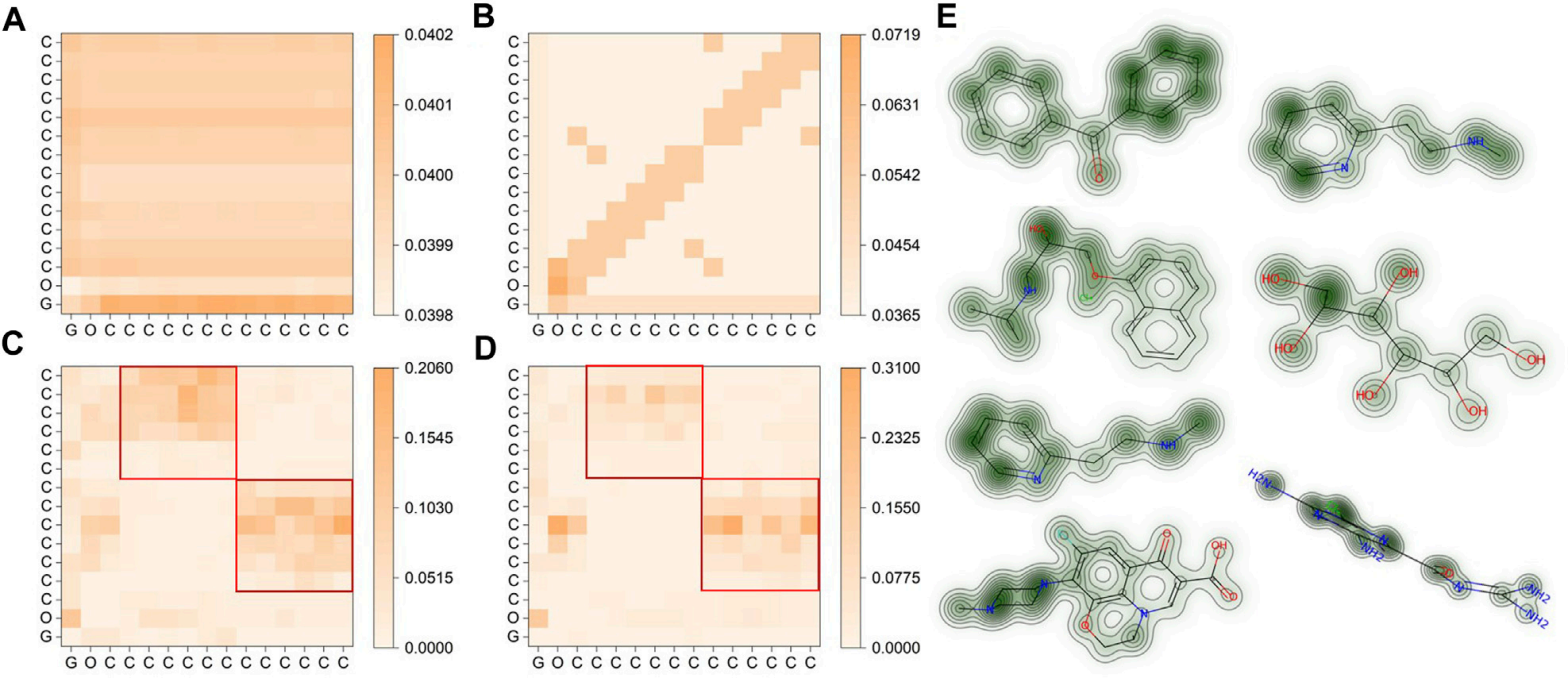
Visualization of molecular representations for benzophenone in SMG-BERT with varying degrees of features. **(A)** Only 1D information is considered. **(B)** 1D + 2D information is considered. **(C)** 1D+ 2D+ 3D geometric information is considered. **(D)** 1D + 2D + 3D + chemical information is considered. The red squares are the positions of the two benzene rings. **(E)** Attention maps for (left column, from top to bottom) benzophenone, propranolol, betahistine, ofloxacin, betahistine, hexitol, and amiloride. Greener areas represent higher weight values.

Figure 3A shows that the molecular representation obtained solely from 1D SMILES string information in pre-training for the benzophenone molecule (depicted in Figure 1) is relatively vague. The similarity between different atoms is within 0.001, indicating a lack of learned explicit chemical information and atomic differences (Figure 3A). However, after incorporating 2D information, the overall correlation between atoms increased, and some regions became more pronounced (Figure 3B). Notably, the current high correlation is closely related to the adjacency matrix, especially the higher attention scores of the atoms themselves, while the correlation in other unrelated regions is relatively low. This suggests that the model initially pays sufficient attention to adjacency information, but it is still not the chemically logical result we expected. Furthermore, the addition of 3D geometric information led to significant changes in the model’s attention scores, with atoms themselves receiving a score of 0 due to the 3D information matrix values, and two nearly symmetrical rectangular regions emerging (Figure 3C). This is because benzophenone has two symmetrical phenyl rings on its left and right sides with nearly identical geometric information. These findings are consistent with expectations and demonstrate that 3D information significantly enhances the model’s output representation, making it more consistent with chemical spatial geometric information. After incorporating the chemical information, noticeable differences are seen in the roughly similar phenyl ring regions compared to the previous results (Figure 3D). This phenomenon could be attributed to the ketone group (C=O), as a strongly polar group, having a stronger electron cloud-attracting ability than the phenyl ring, which disrupts the original large π bond conjugation system of the phenyl ring and re-forms a stable conjugated structure. In this case, the chemical information clearly reflects the influence of the chemical environment on the atoms, such as chemical shifts in NMR. This clearly shows that the added chemical information effectively improves the interpretability of the model and makes the results of the attention matrix more in line with chemical knowledge.

Here we present another six molecules to represent the pre-training results of SMG-BERT (Figure 3E). The model can effectively capture the weight results of different atoms and even differentiate between symmetric substructures in molecules such as benzophenone. Our results highlight the integration of spatial structure information and chemical priors in the model.

### 3.4 Ablation experiment

**FIGURE 4:**
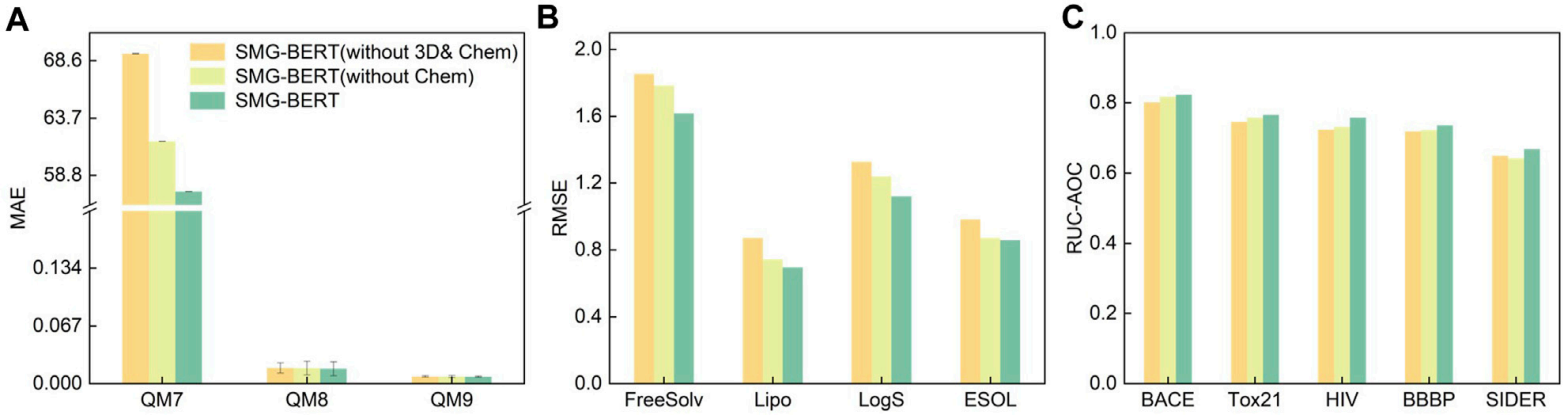
Results of the ablation experiment on regression and classification datasets. Test results on **(A)** QM7, QM8, and QM9 datasets; **(B)** on FreeSolv, Lipo, LogS, and ESOL datasets; and **(C)** on BACE, Tox21, HIV, BBBP, and SIDER datasets.

In this section, we present various ablation analyses of SMG-BERT to gain insight into its remarkable performance. To understand the impact and confirm the importance of explicit information, we performed a series of ablation analyses by removing the corresponding modal components from SMG-BERT. This new variant removes either 3D information and/or chemical information and serves as a comparison to the vanilla version. We conducted 10 random tests on eight datasets for classification and regression tasks. First, we compared the variant without chemical information in terms of changes in classification and regression tasks. Overall, SMG-BERT exhibited varying degrees of performance degradation after removing chemical information, especially in more challenging regression tasks where its RMSE increased by approximately 10% (Figures 4A and 4B). Conversely, removing chemical information had only a small impact on classification tasks, with a decrease of approximately 5% (Figure 4C). This demonstrates that incorporating chemical knowledge can enhance the model’s expressive power and improve its performance. Furthermore, we removed 3D information on this basis (without 3D & Chem) and found that the model’s results became worse, with an average increase in RMSE errors of approximately 7%. This also illustrates the effectiveness and importance of 3D information.

Explicitly adding 3D and chemical information introduces a new problem: an increase in complexity. However, with more complete guidance, unsupervised large-scale models are more likely to learn detailed molecular/atomic features and output precise molecular representations. 3D information increases the model’s attention to the relationship between atoms and unbound atoms, while chemical information supplements the influence of the surrounding groups on atoms. This information can provide guidance for the model’s important domain knowledge, resulting in superior performance. The ablation analysis results of the three sets of experiments undoubtedly confirm the accuracy and robustness of our model. And the importance of 3D and chemical information.

## 4 Conclusion

Molecular representations play an important role in determining both the performance and the interpretability of machine learning models. While most explanatory methods can be applied regardless of the features or descriptors used, the interpretability of features is critical for effective explanations. In particular, features should be both understandable and chemically intuitive whenever possible. For instance, if a specific atom or functional group strongly influences the prediction of high metabolic clearance, a medicinal chemist may consider replacing it. Thus, it is essential that key descriptors are actionable to understand the process by which a prediction is made, which can increase model transparency, facilitate the integration of expert knowledge, enable model tuning for specific applications, and uncover valuable insights, such as learned QSPR patterns.

In this study, we introduced a novel model, called stereo molecular graph BERT (SMG-BERT), which integrates a number of molecular features, including 3D spatial geometric parameters, 2D adjacency information, and 1D SMILES representation, into a self-attention-based BERT architecture. Additionally, SMG-BERT incorporates NMR chemical shifts and BDEs as chemical descriptors through a transformer encoder, which improves interpretability and results in visualizations that are chemically consistent and more compelling As the result shows, SMG-BERT generates accurate chemical representations for various molecules, including chiral molecules, ensuring precise property prediction results and expanding the scope of applications. In contrast, our work focuses exclusively on chiral pairs, meaning that only compounds with a chiral center were considered, while chiral centers in sulfur or phosphorus were excluded. Diastereomers and atropisomers were not taken into account in this work, as diastereomers are not mirror images, and the conformation of atropisomers is typically not described in most activity databases.

## Data Availability

The original contributions presented in the study are included in the article/Supplementary Material, further inquiries can be directed to the corresponding authors.
