# Supplementary material for: SMG-BERT: integrating stereoscopic information and chemical representation for molecular property prediction
Source: Front Mol Biosci. 2023 Jun 30;10:1216765. doi: 10.3389/fmolb.2023.1216765 (PMC10348360; doi:10.3389/fmolb.2023.1216765)
Supplement: Supplementary file 1 [file DataSheet1.PDF]

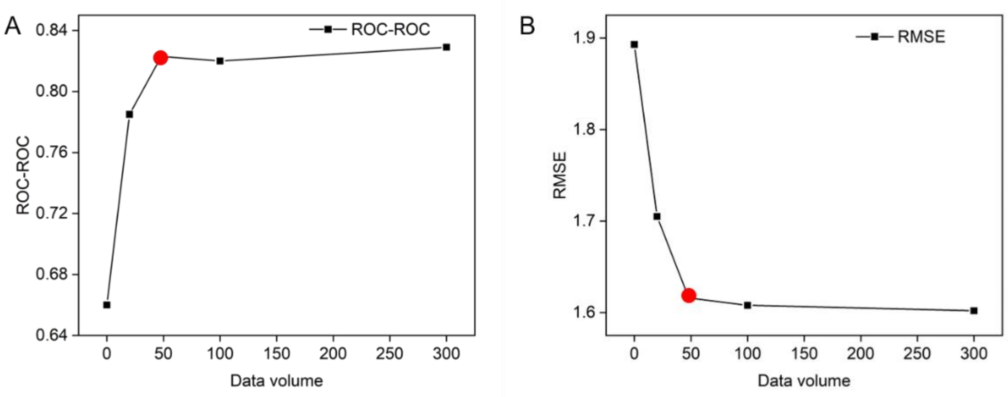

Figure S1. The performance of SMG-BERT under various data size for pretraining (A) is the classification task on BACE dataset and (B) is the regression task on FreeSolv dataset.

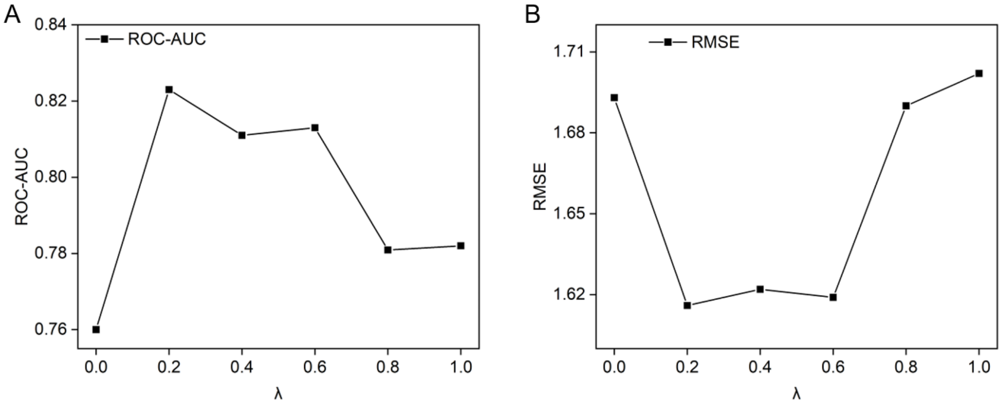

Figure S2. The performance of SMG-BERT under different λ value (A) is the classification task on BACE dataset and (B) is the regression task on FreeSolv dataset.

Table S1： The core pre-training parameters.

optimizer	AdamW
learning rate	0.0001
batch_size	256
embedding dim	128
forward dimension	512
λ	0.2
encoder layer	6
dropout ratio	0.1
epochs	20
mask ratio	0.2

Table S2：The core fine-tuning parameters.

optimizer	AdamW
learning rate	{1e-4,5e-5,1e-5}
batch_size	{16,32}
dropout ratio	{0.0,0.1,0.5}
epochs	100

Table S3. Overall performance for regular regression tasks.

	ESOL	FreeSolv	Lipo	LogS
GIN	77.64(3.82)	78.34(2.32)	67.34(2.32)	83.14(0.92)
GAT	78.12(2.44)	79.20(1.85)	71.23(2.47)	84.09(0.83)
D-MPNN	81.29(1.79)	80.62(1.92)	72.53(2.11)	85.22(0.92)
AttentiveFP	82.87(2.16)	82.25(1.66)	69.32(1.32)	85.25(0.77)
GROVER	84.08(1.73)	84.98(1.42)	74.18(2.02)	84.28(1.22)
GraphMVP	83.93(2.38)	84.23(1.68)	73.43(1.43)	85.23(1.68)
Our Method (no PT)	79.18(2.15)	78.28(2.33)	67.12(2.25)	84.08(1.24)
Our Method (PT)	**85.08**(1.37)	**85.26**(1.29)	**76.12**(0.99)	**86.70**(0.68)

(R^2 is used here. Standard deviations are in brackets; PT: pretraining. Bold number is the best result)

Table S4：The time and GPU running consumption for SMG-BERT and its variants (only 1D+2D).

	Time (per epoch)	GPU memory
SMG-BERT	460s	13628MB
Only 1D+2D	175s	3050MB
